# Clinical and Economic Outcomes of Intravenous Brivaracetam Compared With Levetiracetam for the Treatment of Seizures in United States Hospitals

**DOI:** 10.3389/fneur.2021.760855

**Published:** 2021-11-29

**Authors:** Silky Beaty, Ning A. Rosenthal, Julie Gayle, Prashant Dongre, Kristen Ricchetti-Masterson

**Affiliations:** ^1^UCB Pharma, Smyrna, GA, United States; ^2^Premier Applied Sciences, Premier Inc., Charlotte, NC, United States; ^3^UCB Pharma, Raleigh, NC, United States

**Keywords:** intravenous antiseizure medication, SV2A ligand, seizure, hospital, effectiveness, cost

## Abstract

**Background:** Seizures are common among hospitalized patients. Levetiracetam (LEV), a synaptic vesicle protein 2A (SV2A) ligand, is a common intravenous (IV) anti-seizure medication option in hospitals. Brivaracetam (BRV), a selective SV2A ligand for treatment of focal seizures in patients ≥16 years, has greater binding affinity, higher lipophilicity, and faster brain entry than IV LEV. Differences in clinical outcomes and associated costs between IV BRV and IV LEV in treating hospitalized patients with seizure remain unknown.

**Objectives:** To compare the clinical outcomes, costs, and healthcare resource utilization between patients with seizure treated with IV BRV and those with IV LEV within hospital setting.

**Design/Methods:** A retrospective cohort analysis was performed using chargemaster data from 210 United States hospitals in Premier Healthcare Database. Adult patients (age ≥18 years) treated intravenously with LEV or BRV (with or without BZD) and a seizure discharge diagnosis between July 1, 2016 and December 31, 2019 were included. The cohorts were propensity score-matched 4:1 on baseline characteristics. Outcomes included intubation rates, intensive care unit (ICU) admission, length of stay (LOS), all-cause and seizure-related readmission, total hospitalization cost, and in-hospital mortality. A multivariable regression analysis was performed to determine the association between treatment and main outcomes adjusting for unbalanced confounders.

**Results:** A total of 450 patients were analyzed (IV LEV, *n* = 360 vs. IV BRV, *n* = 90). Patients treated with IV BRV had lower crude prevalence of ICU admission (14.4 vs. 24.2%, *P* < 0.05), 30-day all-cause readmission (1.1 vs. 6.4%, *P* = 0.06), seizure-related 30-day readmission (0 vs. 4.2%, *P* < 0.05), similar mean total hospitalization costs ($13,715 vs. $13,419, *P* = 0.91), intubation (0 vs. 1.1%, *P* = 0.59), and in-hospital mortality (4.4 vs. 3.9%, *P* = 0.77). The adjusted odds for ICU admission (adjusted odds ratio [_a_OR] = *0.6*; 95% confidence interval [CI]:0.31, 1.16; *P* = 0.13), 30-day all-cause readmission (_a_OR = 0.17; 95% CI:0.02, 1.24; *P* = 0.08), and in-hospital mortality (_a_OR = 1.15; 95% CI:0.37, 3.58, *P* = 0.81) were statistically similar between comparison groups.

**Conclusion:** The use of IV BRV may provide an alternative to IV LEV for management of seizures in hospital setting due to lower or comparable prevalence of ICU admission, intubation, and 30-day seizure-related readmission. Additional studies with greater statistical power are needed to confirm these findings.

## Introduction

As the most frequent clinical presentation of epilepsy, a chronic condition of the brain, seizures are associated with substantial psychological, physical, and economic burden on patients and caregivers (1–3). Seizures may affect people of all ages and geographical regions, and are especially common among patients in the hospital setting (4). Seizures are prevalent across the hospital with rates varying by setting. Specifically, seizures account for about 1% (1 million visits annually) of all emergency department (ED) visits (4). According to the Agency for Healthcare Research and Quality's Statistical Brief #45, seizures or epilepsy were identified in ~3.6% of a total of 39.2 million hospitalizations in 2005 (5). Within the intensive care unit (ICU), about 10% of patients experience seizures; this rate increases to as much as 33% in the neurocritical care unit (NCCU) (6, 7).

Focal seizures (both convulsive and non-convulsive types) occur commonly in the ICU setting. Seizures amongst critically ill patients in the ICU setting are associated with poor outcomes especially among those with structural brain lesions (e.g., brain tumors, traumatic brain injury, and stroke, including both ischemic and hemorrhagic stroke) (8). In a study on 402 patients with subarachnoid hemorrhage treated in a single medical center NCCU, De Marchis et al. showed that every hour of seizure on a continuous EEG during the inpatient stay was associated with 10% increased odds of 3-month disability and mortality (9). In addition, seizures are often recurrent in the hospital setting. According to findings from the study of Fields et al. nearly two-thirds of hospitalized patients with new-onset seizure had a second seizure during their inpatient stay and over one-fifth of the recurrent seizures occurred on the first day and 39% occurred on more than 1 day (10). Beyond the management of status epilepticus, there is shortage of evidence-based recommendations for the management of seizures within the hospital setting among critically ill patients that aim to improve patient outcomes.

Among the many anti-seizure medications (ASMs) available in the United States (US), less than a third are available in an intravenous (IV) formulation to offer rapid availability when oral formulations may not be feasible for acute seizure management in the ICU setting (11). The most recently approved ASM available in an IV formulation, brivaracetam (BRV), a selective high-affinity ligand for synaptic vesicle protein 2A (SV2A), was originally approved by the US Food and Drug Administration in 2016 and is currently indicated as monotherapy or adjunctive treatment for partial-onset (focal) seizures (POSs) in patients aged 1 month and older. BRV is also approved in Europe and other regions (12). In addition to being administered through an intravenous infusion, IV BRV is approved for administration *via* a 2-min bolus injection without dilution, making it a useful option for the treatment of POS in hospital setting. Compared with levetiracetam (LEV), the first approved ASM targeting SV2A, BRV has 15- to 30-fold greater binding affinity, high lipophilicity, and faster brain entry (13, 14). Physicians treating patients in ICU and NCCU constantly face difficulties of selecting appropriate IV ASMs from the few treatment options available to maintain seizure control. There are limited real-world data that compare clinical outcomes, costs, and healthcare resource utilization (HRU) for all IV ASMs, especially between two ASMs of the same drug class (15–17).

This study aimed to fill the gap and compare the clinical outcomes, costs, and HRU of monotherapy of IV BRV with IV LEV, with or without benzodiazepine (BZD), for the treatment of seizures within the US hospital setting. Such information will provide a much-needed reference for physicians to handle seizures appropriately in acute care setting especially in ICU/NCCU.

## Methods

### Study Design, Setting, and Participants

A retrospective observational study was conducted to address the study objectives among adult patients (age ≥18 years) diagnosed with seizures (having principal or secondary international classification of disease version 10 with clinical modification [ICD-10-CM] diagnosis codes for seizures, as shown in Supplementary Table 1) and treated with monotherapy IV BRV or IV LEV, with or without benzodiazepine (BZD) use during an inpatient hospital visit between July 1, 2016 and December 31, 2019. Patients from hospitals with no continuous data submission during the 90-day look-back and 30-day follow-up periods were excluded from the analysis. Propensity score matching with Mahalanobis distance methods was performed to match patients with IV BRV use and those with IV LEV use by a 1:4 ratio. Variables included in the logistic regression model for generating the propensity score were sex, race, primary payer type, hospital urban/rural status, teaching/non-teaching status, hospital bed size, type of seizure diagnosis (principal vs. secondary), source of admission, hypertension, and cardiac arrythmia. Model variable selection was based on prior knowledge and significant differences in distribution of these variables between comparison groups shown in this analysis and potential association between these variables and the outcomes.

### Study Variables

The major exposure variable was status of IV BRV and IV LEV use among patients with a discharge diagnosis of seizure for their index inpatient visit. Medication use status including route of administration was determined by searching chargemaster descriptions with specific drug names and route of administration.

Primary outcomes of interest included in-hospital mortality, total length of stay (LOS), and total costs during index hospitalization as well as risks of all-cause readmission and seizure-related readmission during 30 days post index hospitalization. Secondary outcomes included intubation, ICU admission, ICU LOS, and selected departmental costs during index hospitalization.

Other covariates assessed included patient demographic characteristics (i.e., age, sex, race, ethnicity, payer type), hospital characteristics (i.e., population served, teaching status, geographical region, and hospital size), admission point of origin, admission type, discharge status, and baseline comorbidities. St. Germaine-Smith's seizure-specific comorbidities were assessed during both the index hospitalization and hospital visits during 6 months prior to the index admission (18). The seizure-specific comorbidity scale included congestive heart failure, peripheral vascular disease, renal disease, moderate to severe liver disease, metastatic cancer, brain tumor, solid tumor without metastasis, paraplegia and hemiplegia, aspiration pneumonia, dementia, pulmonary circulation disease, cardiac arrhythmias, hypertension, and anoxic brain injury (see Supplementary Table 2 for comprehensive list of comorbid conditions and ICD-10 codes).

### Data Source

Data used for this study were derived from the Premier Healthcare Database (PHD), a large geographically diverse hospital-based, service-level, and all-payer database containing discharge information from inpatient and hospital-based outpatient visits (12). It has been representing ~20–25% of all inpatient admissions in the US since 2000. As of June 1, 2020, there were over one billion inpatient and outpatient discharges from 1,057 hospitals included in the PHD. All the data are statistically de-identified and compliant with the Health Insurance Portability and Accountability Act. Patients can be tracked within the hospital through a unique identifier. The PHD contains patient and visit-level data from standard hospital discharge files such as patient demographics, disease states, and time-stamped log of billed items, namely, procedures, medications, laboratory, and diagnostic and therapeutic services. Information on hospital geographic location, rural/urban populations served, teaching status, and bed capacity is available. Institutional review board (IRB) approval for this study was not required, based on US Title 45 Code of Federal Regulations, Part 46, because the study used existing de-identified hospital discharge data, and recorded information could not be identified directly or through identifiers linked to individuals. No informed consent of study participants was pursued because of the nature of the de-identified data.

### Bias

Even though robust validation measures have been taken, miscoding or underreporting of ICD-10 diagnosis codes may result in misclassification of covariates. However, such misclassifications shall be non-differential between the two comparison groups. Selection bias may also exist because the PHD only captures 20–25% of all inpatient visits in the US. Patients in the two treatment groups may be different from all patients treated with IV BRV and IV LEV in the country. The impact of such potential selection bias on outcomes remains unknown.

### Study Size

All patients with seizure who met the selection criteria were included in the initial patient cohort that included 94 patients with IV BRV and 228, 431 with IV LEV. During 1 to 4 propensity score matching, we failed to find matches for four patients in the IV BRV group, which resulted in a final sample of 90 IV BRV patients.

### Missing Data and Outliers

None of the study variables for this cohort had missing data. Outliers were observed for LOS and cost values. Winsorization of patient records above the 99th percentile (values above the 99th percentile was replaced by values at 99th percentile) and below the 1st percentile was performed to account for extremely high or low LOS and cost values based on the actual distribution of such data in this study (19, 20).

### Statistical Analysis

We first examined the distribution of all outcomes, exposures, and covariates, and checked for missing data and outliers. Data measured on a continuous scale was expressed as mean, standard deviation, median, and interquartile range (IQR). Categorical data were expressed as counts and percentages of patients in the categories. Then, patient characteristics and main outcome variables were reported by treatment regimen (IV BRV vs. IV LEV). Chi-square or Fisher's tests were performed to test for statistical differences between groups for categorical variables. Two sample comparisons were evaluated by *t*-test or Wilcoxon Rank Sum test for continuous variables.

A multivariable regression analysis was performed to assess the association between treatment regimen and key outcome variables adjusting for potential confounding variables that remained unbalanced between comparison groups post propensity score matching, such as hospital region, hospital bed size, and dementia status. For total index inpatient visit cost analysis, generalized linear regression modeling with log link and gamma distribution was performed to compare differences in cost between treatment groups. Adjusted mean and 95% CI from the generalized linear modeling results were reported. Multivariable logistic regression was performed to assess the difference in all-cause in-hospital mortality and 30-day readmission risk between treatment groups. Multivariable negative binomial modeling was performed to compare differences in total hospital LOS between treatment groups.

Statistical significance level or alpha is set to 0.05. All the analyses were conducted using SAS 9.4 Statistical Software.

## Results

### Patient and Hospital Characteristics

A total of 360 patients with IV LEV from 206 hospitals and 90 patients with IV BRV from 23 hospitals were included in the analysis. After matching, the distribution of age, sex, race, ethnicity, primary payer type, hospital population served, and teaching status were comparable between the IV LEV and IV BRV treatment groups. The mean age of the study cohort was 44 ± 17years, with more than a third (35.6%) aged 18 to 34 years. Approximately half of the overall population (51.6%) were male, 70.2% were White, 28% were commercially insured, 33.1% were insured by Medicare, and 33.6% were insured by Medicaid. The majority of patients (96.2%) were from urban hospitals and 54.4% were from teaching hospitals. For hospital region, patients with IV BRV were more heavily concentrated in the South (75.6%) compared with patients with IV LEV (48.1%). A higher percentage of patients with IV LEV were from the Midwest (23.6 vs. 13.3%), Northeast (18.9 vs. 6.7%), and West (9.4 vs. 4.4%) than patients with IV BRV (*P* = 0.01). A higher percentage of patients with IV LEV were treated in hospitals with 500+ beds than patients with IV BRV (*P* < 0.01) (Table 1).

**Table 1:** Patient demographic and hospital characteristics by treatment status.

**Variables**	**Overall**		IV LEV^*^		IV BRV^**^		**P-value**
	***N***	**%**	***N***	**%**	***N***	**%**	
**# of unique patients**	450		360		90		
**# of hospitals**	210		206		23		
**Mean age (Std, years)** ^**a**^	44 ± 17		44 ± 17		44 ± 17		
**Age group (years)**							1.00
18–34	160	35.60%	128	35.60%	32	35.60%	
35–49	125	27.80%	100	27.80%	25	27.80%	
50–64	95	21.10%	76	21.10%	19	21.10%	
65–74	50	11.10%	40	11.10%	10	11.10%	
75+	20	4.40%	16	4.40%	4	4.40%	
**Sex** ^**a**^							0.92
Male	232	51.60%	186	51.70%	46	51.10%	
Female	218	48.40%	174	48.30%	44	48.90%	
**Race** ^**a**^							0.94
White	316	70.20%	252	70.00%	64	71.10%	
Black	101	22.40%	82	22.80%	19	21.10%	
Other	33	7.30%	26	7.20%	7	7.80%	
**Ethnicity**							0.47
Hispanic	34	7.60%	30	8.30%	4	4.40%	
Non-Hispanic	316	70.20%	244	67.80%	72	80.00%	
Unknown	100	22.20%	86	23.90%	14	15.60%	
**Primary payer** ^**a**^							0.93
Commercial	126	28.00%	103	28.60%	23	25.60%	
Medicare	149	33.10%	117	32.50%	32	35.60%	
Medicaid	151	33.60%	121	33.60%	30	33.30%	
Other Payor	24	5.30%	19	5.30%	5	5.60%	
**Hospital population served** ^**a**^							0.76
Urban	433	96.20%	347	96.40%	86	95.60%	
Rural	17	3.80%	13	3.60%	4	4.40%	
**Hospital teaching status** ^**a**^							0.08
Teaching	245	54.40%	204	56.70%	41	45.60%	
Non-Teaching	205	45.60%	156	43.30%	49	54.40%	
**Hospital census regions**							0.01
Midwest	97	21.60%	85	23.60%	12	13.30%	
Northeast	74	16.40%	68	18.90%	6	6.70%	
South	241	53.60%	173	48.10%	68	75.60%	
West	38	8.40%	34	9.40%	4	4.40%	
**Hospital bed size** ^**a**^							0.003
<100	12	2.70%	11	3.10%	1	1.10%	
100–199	48	10.70%	36	10.00%	12	13.30%	
200–299	72	16.00%	55	15.30%	17	18.90%	
300–499	139	30.90%	99	27.50%	40	44.40%	
500+	179	39.80%	159	44.20%	20	22.20%	

* *IV LEV, Intravenous Levetiracetam*;

** *IV BRV, Intravenous Brivaracetam*.

a *Variables included in the propensity score matching*.

Over one-third of patients in each treatment group had IV BZD use (38.9% for IV BRV and 34.7% for the IV LEV group, *P* = 0.46). The distribution of different types of seizures varied between patients with IV LEV and those with IV BRV, with higher percentages of IV BRV patients having a specified diagnosis of focal seizures (POS) (37.8 vs. 16.1%, respectively) or generalized seizures (23.3 vs. 15.3%, respectively) compared with patients with IV LEV (*P*< *0.0*1). There was no statistically significant difference in the prevalence of status epilepticus, discharge status, and mean baseline comorbidity index score between patients treated with IV LEV and those treated with IV BRV, as seen in Table 2. Nearly two-thirds of the patients (63.3%) were admitted through ED, 73.8% were discharged home, 10.4% were transferred to another acute care hospital, and 78.9% had epilepsy as their principal diagnosis for the index hospitalization. The mean comorbidity index score was 1.19 ± 2.16 for patients with IV LEV and 0.88 ± 1.59 for patients with IV BRV (*P* = 0.2). The most common comorbidity was hypertension (27.8%), followed by cardiac arrhythmias (8.9%), congestive heart failure (6.7%), and peripheral vascular disease (4.7%). The prevalence of dementia was significantly higher among patients with IV BRV compared with those with IV LEV (11.1 vs. 2.5%, *P* < 0.01). Brain tumor and anoxic brain injury accounted for 2.4 and 3.3% of patients overall, respectively, with no statistical difference between treatment groups (Table 2).

**Table 2:** Patient clinical characteristics by treatment status.

	**Overall (*****n*** **=** **450)**		IV LEV^*^ **(*****n*** **=** **360)**		IV BRV^**^ **(*****n*** **=** **90)**		***P*-value**
	***N***	**%**	***N***	**%**	***N***	**%**	
**Type of seizure**							
Partial Onset Seizure	92	20.40%	58	16.10%	34	37.80%	<0.001
Generalized Seizure	76	16.90%	55	15.30%	21	23.30%	
Other Seizure	151	33.60%	138	38.30%	13	14.40%	
Unspecified Seizure	131	29.10%	109	30.30%	22	24.40%	
**Had status epilepticus**							
Yes	32	7.10%	25	6.90%	7	7.80%	0.78
No	418	92.90%	335	93.10%	83	92.20%	
**Admission source** ^**a**^							
Emergency	285	63.30%	228	63.30%	57	63.30%	1.00
Physician Referral	165	36.70%	132	36.70%	33	36.70%	
**Discharge status**							
Expired	18	4.00%	14	3.90%	4	4.40%	0.55
Home	332	73.80%	264	73.30%	68	75.60%	
Hospice	8	1.80%	7	1.90%	1	1.10%	
Skilled nursing facility or Long-Term Care	33	7.30%	23	6.40%	10	11.10%	
Transferred to another acute care hospital	47	10.40%	41	11.40%	6	6.70%	
Other	12	2.70%	11	3.10%	1	1.10%	
**Seizure diagnosis type** ^**a**^							
Principal	355	78.90%	284	78.90%	71	78.90%	1.00
Secondary	95	21.10%	76	21.10%	19	21.10%	
**IV Benzodiazepine use**	160	35.60%	125	34.70%	35	38.90%	0.46
**Baseline comorbidity**							
Congestive heart failure	30	6.70%	23	6.40%	7	7.80%	0.64
Peripheral vascular disease	21	4.70%	16	4.40%	5	5.60%	0.59
Renal disease	12	2.70%	12	3.30%	0	0.00%	0.14
Moderate or severe liver disease	5	1.10%	5	1.40%	0	0.00%	0.59
Metastatic cancer	16	3.60%	15	4.20%	1	1.10%	0.21
Brain tumor	11	2.40%	9	2.50%	2	2.20%	1
Solid tumor without metastasis	18	4.00%	16	4.40%	2	2.20%	0.55
Paraplegia and hemiplegia	1	0.20%	0	0.00%	1	1.10%	0.2
Aspiration pneumonia	18	4.00%	15	4.20%	3	3.30%	1
Dementia	19	4.20%	9	2.50%	10	11.10%	0.0012
Pulmonary circulation disorders	7	1.60%	6	1.70%	1	1.10%	1
Cardiac arrhythmias^a^	40	8.90%	32	8.90%	8	8.90%	1
Hypertension^a^	125	27.80%	100	27.80%	25	27.80%	1
Anoxic brain jnjury	15	3.30%	13	3.60%	2	2.20%	0.75
**Comorbidity index score (**Mean-Std Dev)	1.13	±2.06	1.19	±2.16	0.88	±1.59	0.2

* *LEV, Levetiracetam*;

** *BRV, Brivaracetam*.

a *Variables included in the propensity score matching*.

### Unadjusted Results for Primary and Secondary Outcomes

As shown in Table 3, the unadjusted in-hospital mortality rate during index hospitalization was 3.9% for patients with IV LEV and 4.4% for patients with IV BRV (*P* = 0.77). The median LOS for index hospitalization was 3 days for both IV LEV (IQR: 1, 4) and IV BRV (IQR: 2, 4) (*P* = 0.38). The median index hospitalization costs were also comparable between patients with IV LEV (median: $6,304; 25–75th percentiles: $2,186, $13,180) and those with IV BRV (median: $6,261; 25–75th percentiles: $4,076, $11,182) (*P* = 0.91). Patients with IV LEV tended to have higher risk of 30-day all-cause readmission (6.4 vs. 1.1%, *P* = 0.06, and 4.2% of IV LEV patients had seizure-related readmission, while patients with IV BRV did not have any seizure-related readmissions observed during the 30 days post discharge from the index hospitalization (*P* = 0.05).

**Table 3:** Unadjusted results for primary and secondary outcomes by treatment status.

	**Overall (*****n*** **=** **450)**		IV LEV^*^ **(*****n*** **=** **360)**		IV BRV^**^ **(*****n*** **=** **90)**		***P*-value**
	***n***	**%**	***n***	**%**	***n***	**%**	
**PRIMARY OUTCOMES**							
**In-hospital mortality**	18	4.00%	14	3.90%	4	4.40%	0.77
**Total length of stay (days)**							0.38
Mean-Std Dev	3.9	±5.6	3.79	±5.81	4.3	±4.72	
Median (IQR^#^)	3	(1, 4)	3	(1, 4)	3	(2, 4)	
**Total index hospitalization cost (in 2019 U.S. dollars)**							0.91
Mean-Std Dev	13,478	±22,282	13,419	±22,058	13,715	±23,282	
Median	6,284		6,304		6,261		
IQR^#^	2,563	12,961	2,186	13,180	4,076	11,182	
**30-day all-cause readmission**	24	5.30%	23	6.40%	1	1.10%	0.06
**30-day seizure-related readmission**	15	3.30%	15	4.20%	0	0.00%	0.05
**SECONDARY OUTCOMES**							
**Intubation**	4	0.90%	4	1.10%	0	0%	0.58
**ICU admission** ^**a**^	100	22.20%	87	24.20%	13	14.40%	0.05
**NCCU admission** ^**a**^	2	0.40%	2	0.60%	0	0.00%	1.00
**ICU length of stay (days)** ^**a**^							0.35
Mean-Std Dev	4.9	±5.85	4.64	±5.65	6.62	±7.05	
Median	3		2		3		
IQR^#^	1	6.5	1	5	2	9	
**ICU Costs (in 2019 U.S. Dollars)** ^**a**^							0.51
Mean-Std Dev	26425.34	±29681.89	25254.06	±26296.66	34263.9	±47286.45	
Median	17891.87		18096.62		16046.79		
IQR^#^	8281.46	33431.28	8407.15	32239.94	6527.22	3,5957	
**Room and board costs (in 2019 U.S. dollars)**							0.5
Mean-Std Dev	8058.93	±10786.68	8289.68	±10519.07	7270.03	±11696.75	
Median	4458.7		4793.29		3870.23		
IQR^#^	2702.56	8685.06	2714.19	9529.61	2628.6	6691.56	
**Pharmacy costs (in 2019 U.S. dollars)**							0.31
Mean-Std Dev	1383.22	±3432.49	1262.13	±2725.38	1907.47	±5533.07	
Median	424.69		413.71		448.32		
IQR^#^	177.89	996.63	165.78	1026.13	226.64	859.78	

* *IV LEV, Intravenous Levetiracetam*;

** *IV BRV, Intravenous Brivaracetam*;

# *IQR, interquartile range*.

a *ICU = intensive care unit; NCCU = neurocritical care unit; NCCU admission is a subset of all ICU admissions*.

For secondary outcomes, patients with IV LEV had higher prevalence of ICU admission than those with IV BRV (24.2 vs. 14.4%, *P* = *0.0*5), but the two treatment groups had similar intubation rate, ICU LOS, ICU costs, room and board costs, and pharmacy costs (Table 3).

### Adjusted Results for Primary and Secondary Outcomes

After adjusting for unbalanced covariates, such as dementia status, hospital region, and hospital bed size, no statistical difference was observed in total hospitalization cost (Adjusted mean [95% CI]: $12,993 [$10,025, $16,839] in IV BRV vs. $12,564 [$11,140, $14,171] in IV LEV, *P* = 0.8249), ICU cost, total LOS, ICU stay, and in-hospital mortality between patients with use of IV LEV and IV BRV. The adjusted room and board cost was $2,406 higher for patients with IV LEV than those with IV BRV (*P* = *0.0*1), while the adjusted pharmacy cost was $505 lower for patients with IV LEV than those with IV BRV (*P* = *0.0*6). The adjusted odds of having 30-day all-cause readmission were 83% lower in the IV BRV group than in the IV LEV group (adjusted odds ratio [_a_OR]: 0.17; 95% confidence interval [CI]: 0.02, 1.24, *P* = 0.08). The adjusted odds of ICU admission were 40% lower among patients with IV BRV compared with those with IV LEV (_a_OR: 0.6, 95% CI: 0.31, 1.16, *P* = 0.13), but it was not statistically significant (Table 4).

**Table 4:** Multivariable regression results for outcomes by treatment status.

**Outcome variables**	IV LEV^*^	IV BRV^**^	***P*-value**
**Total index**			
**hospitalization cost**			
Adjusted mean	$12,564	$12,993	0.82
95% CI^**a**^	$11,140, $14,171	$10,025, $16,839	
**Total ICU Cost** ^**a**^			
Adjusted mean	$24,566	$30,480	0.56
95% CI^**a**^	$20,103, $30,020	$15,650, $59,362	
**Room and board cost**			
Adjusted mean	$8,331	$5,925	0.01
95% CI^**a**^	$7,415, $9,360	$4,762, $7,371	
**Pharmacy Cost**			
Adjusted mean	$1,262	$1,767	0.057
95% CI Range^**a**^	$1,097, $1,451	$1,298, $2,404	
**Total LOS** ^**a**^			
Adjusted mean	3.63	4.13	0.38
95% CI	3.21, 4.11	3.21, 5.32	
**ICU LOS** ^**a**^			
Adjusted mean	4.56	4.34	0.88
95% CI^**a**^	3.72, 5.58	2.39, 7.89	
		**OR (95% CI)**	**P-value**
**In-hospital mortality**	Reference	1.15 (0.37, 3.58)	0.81
**30-day all-cause readmission**	Reference	0.17 (0.02, 1.24)	0.08
**ICU admission** ^**a**^	Reference	0.6 (0.31, 1.16)	0.13

*Notes: Multivariable generalized linear model with log link and gamma distribution was used for cost variables. Multivariable generalized linear model with log link and negative binomial model was used for length of stay variables. Multivariable logistic regression was used for in-hospital mortality, 30-day all-cause readmission and ICU admission. Covariates adjusted in the models include dementia status, hospital region and hospital bed size*.

* *IV LEV, Intravenous Levetiracetam*;

** *IV BRV, Intravenous Brivaracetam*.

a *ICU, intensive care unit; LOS, length of stay; CI, confidence interval*.

## Discussion

This real-world evidence study represents one of the first efforts to evaluate the clinical outcomes, costs, and HRU in patients with seizure receiving IV BRV or IV LEV (the only other ASM currently in the same class) in hospital setting. The findings of this study provide valuable information to facilitate clinical decision-making in seizure treatment in hospital, including ICU/NCCU setting. The results from this study showed that there was no statistically significant difference in ICU LOS, total hospital LOS, in-hospital mortality, total hospitalization costs, and ICU cost between the IV LEV and IV BRV groups in both unadjusted and adjusted analyses. However, the unadjusted 30-day seizure-related readmission risk and prevalence of ICU admission were significantly higher in IV LEV than in IV BRV. No patient in the IV BRV group had intubation during index hospitalization compared with 1.1% of patients with IV LEV, although the difference is not statistically significant. The high prevalence of ICU admission indicated that patients on IV LEV or BRV are often severe. Among the study population, IV BRV showed promising results for reducing 30-day readmission risks without incurring higher cost. The costs assessed for the period of this analysis were prior to BRV discounts that became effective in January of 2020.

The patients on IV BRV or LEV included in this study were relatively young, with the majority being White and non-Hispanic. Insurance coverage was evenly distributed across all patients. Nearly two-thirds of the patients were admitted through ED with a principal diagnosis of seizure in over three-quarters of patients across all settings within the hospital. Over one-fifth of the patients (22%) were admitted to ICU, over 10% were transferred to another acute care facility, and 4% of the patients died during index hospitalization. Baseline comorbidities identified in visits to the same hospital system with the index hospitalization were not very common, with a mean comorbidity index score of 1.13 ± 2.06, possibly due to younger age of the IV BRV patients and matched controls. The demographics of the patients are similar to what was reported in the analysis by Pallin of seizure-related ED visits, in which 66% of adult patients were between 20 and 50 years of age, 68% were White, and 74% were Non-Hispanic (4). Comorbid conditions, such as hypertension (1.4%) and cerebrovascular disease (0.8%), were uncommon in the ED sample (4).

Although no direct clinical outcome comparisons between IV LEV and IV BRV have been made in prior literature, independent studies have shown that IV BRV and IV LEV might be efficacious and tolerable in patients with acute seizures in the hospital setting (21–27). IV BRV 100 and 200 mg had similar time to next seizure as IV lorazepam in patients with acute seizure activity admitted in epilepsy monitoring unit (EMU) as evaluated in a small pilot study (28). Although we do not have a proven causative linkage between seizure treatment and improvement in clinical outcomes, it has been proposed that seizures could fundamentally damage the brain and could potentially lead to increased rates of intubation, ICU admission, and increased length of stay in ICU and hospital, leading to worsening of outcomes and increased costs (29). Hence, prompt and appropriate treatment of seizures may decrease these complications and prevent poor clinical outcomes (30). IV levetiracetam is one of the commonly used ASMs for new-onset seizure in hospitals and in critical care patients (31). IV BRV has shown good clinical tolerability, efficacy, and favorable pharmacokinetic profile (12, 13, 21, 28). Proton-emission tomography (PET) imaging studies conducted on healthy human volunteers provided direct clinical evidence that BRV enters the brain faster than LEV, consistent with previous preclinical data. More rapid penetration of BRV vs. LEV provides the potential for more rapid onset of action and, therefore, could be important in acute seizures requiring prompt therapeutic intervention that can be further confirmed in prospective clinical studies (13). The analysis of the propensity score matched data in the present retrospective study shows that IV BRV had similar or, in some cases, numerically better clinical outcomes than IV LEV.

No identified study has directly compared the HRU and cost outcomes between IV LEV and IV BRV. Despite the large database from over 210 US hospitals that comprises both partial and generalized seizures with and without status epilepticus, the sample of IV BRV patients remains limited. However, we were able to demonstrate in the propensity score-matched analysis that patients treated with IV BRV had similar in-hospital mortality rate, total hospital length of stay, and costs during the index hospitalization with patients treated with IV LEV. Of note, total hospital costs remained similar after adjustment between IV BRV and IV LEV, despite the higher perceived pharmaceutical cost for IV BRV. None of the patients treated with IV BRV had intubation during index hospitalization despite similar use of benzodiazepines between the IV BRV and matched IV LEV patients or had seizure-related readmission during 30 days post index hospitalization compared with 1.1 and 4.2% among those with IV LEV. Furthermore, IV BRV was also associated with lower adjusted odds of ICU admission than those with IV LEV; however, more research is needed to establish the temporal relationship. These findings imply that IV BRV could be a faster alternative to IV LEV in treating seizures in hospital setting, due to the approved 2-min bolus and rapid permeability across the blood-brain barrier.

This study has multiple strengths. First, the PHD is the largest hospital administrative database in the US and covers one in four to five hospital inpatient discharges in the nation across 45 states with date-stamped services provided in hospital setting. The database provides a representative sample to address the core research questions. Second, the PHD captures detail medications used in hospital setting, which allowed us to comprehensively assess the treatment patterns for seizure patients. Third, cost data in PHD are submitted by hospitals to reflect the actual cost of each service to the hospitals, which provides more accurate estimate of burden to the hospitals than charges or reimbursement amount.

The study also has several limitations. The PHD only captures visits to the same hospital for each patient. The readmission risk might be underestimated. However, the level of underestimation shall be non-differential between comparison groups. In addition, seizures are determined by ICD-10 diagnosis codes. Underreporting or miscoding could exist. The estimates on these conditions may be underestimated. As with the first limitation, the underreporting or miscoding may be non-differential between the comparison groups. Furthermore, there may be other factors affecting use of medications in the comparison that are not captured by the database, such as clinical preference or specific disease state, which may result in confounding by indication. Lastly, due to the lack of information on timing of treatment within the hospital, the associations presented are not evidence of causality.

In conclusion, this propensity score-matched cohort study using a nationally representative sample of patients with seizure demonstrated that patients treated with IV BRV have lower prevalence of ICU admission and risk of 30-day seizure-related readmissions when compared with IV LEV patients. Total hospitalization cost and cost of ICU stay were not statistically different between IV BRV and IV LEV. Based on these trends, we conclude that the use of IV BRV may provide a good alternative to IV LEV for the management of seizures in the hospital setting. A well-designed prospective randomized study with an adequate sample size would be beneficial to confirm our findings.

## Data Availability Statement

The data analyzed in this study was obtained from the Premier Healthcare Database, a proprietary HIPAA-compliant de-identified database, the following licenses/restrictions apply: requests to access the de-identified datasets must first be approved. Requests to access these datasets should be directed to Ning Rosenthal, ning_rosenthal@premierinc.com.

## Ethics Statement

Ethical review and approval was not required for the study on human participants in accordance with the local legislation and institutional requirements. Written informed consent for participation was not required for this study in accordance with the national legislation and the institutional requirements.

## Author Contributions

All authors listed have made a substantial, direct, and intellectual contribution to the work and approved it for publication.

## Conflict of Interest

SB, PD, and KR are employees of UCB Inc. NR and JG are employees of Premier Inc.

## Publisher's Note

All claims expressed in this article are solely those of the authors and do not necessarily represent those of their affiliated organizations, or those of the publisher, the editors and the reviewers. Any product that may be evaluated in this article, or claim that may be made by its manufacturer, is not guaranteed or endorsed by the publisher.

## References

[1] Beghi E. The epidemiology of epilepsy. Neuroepidemiology. (2020) 2020:185–91. 10.1159/000503831 31852003

[2] Hussain SA Ortendahl JD Bentley TG Harmon AL Gupta S Begley CE. The economic burden of caregiving in epilepsy: an estimate based on a survey of US caregivers. Epilepsia. (2020) 2020:319–29. 10.1111/epi.16429 31953846

[3] Jafarpour S Hirsch LJ Gaínza-Lein M Kellinghaus C Detyniecki K. Seizure cluster: definition, prevalence, consequences, and management. Seizure. (2019) 68:9–15. 10.1016/j.seizure.2018.05.013 29871784

[4] Pallin DJ Goldstein JN Moussally JS Pelletier AJ Green AR Camargo CA. Seizure visits in US emergency departments: epidemiology and potential disparities in care. Int J Emerg Med. (2008) 1:97–105. 10.1007/s12245-008-0024-4 19384659 PMC2657249

[5] Holmquist L Russo CA Elixhauser A. Hospitalizations for Epilepsy and Convulsions, 2005: Statistical Brief #46, in Healthcare Cost and Utilization Project (HCUP) Statistical Briefs 2006. Rockville, MD: Agency for Healthcare Research and Quality (US) (2006). 21755631

[6] Fogang Y Legros B Depondt C Mavroudakis N Gaspard N. Yield of repeated intermittent EEG for seizure detection in critically ill adults. Neurophysiol Clin. (2017) 47:5–12. 10.1016/j.neucli.2016.09.001 27771198

[7] Schmitt SE. Utility of clinical features for the diagnosis of seizures in the intensive care unit. J Clin Neurophysiol. (2017) 2017:158–61. 10.1097/WNP.0000000000000335 27571047

[8] Aronica E Mühlebner A. Neuropathology of epilepsy. Handb Clin Neurol. (2017) 145:193–216. 10.1016/B978-0-12-802395-2.00015-8 28987170

[9] De Marchis GM Pugin D Meyers E Velasquez A Suwatcharangkoon S Park S. Seizure burden in subarachnoid hemorrhage associated with functional and cognitive outcome. Neurology. (2016) 86:253–60. 10.1212/WNL.0000000000002281 26701381 PMC4733156

[10] Fields MC Labovitz DL French JA. Hospital-onset seizures: an inpatient study. JAMA Neurol. (2013) 70:360–4. 10.1001/2013.jamaneurol.337 23319087

[11] Vossler DG Weingarten M Gidal BE. Summary of Antiepileptic Drugs Available in the United States of America: working toward a world without epilepsy. Epilepsy Curr. (2018) 18(4 Suppl 1):1–26. 10.5698/1535-7597.18.4s1.1 30233275 PMC6130739

[12] Agency EM. Briviact (2018). Available online at: https://www.ema.europa.eu/en/medicines/human/EPAR/briviact-italy-nubriveo. (accessed April, 2021)

[13] Finnema SJ Rossano S Naganawa M Henry S Gao H Pracitto R. A single-center, open-label positron emission tomography study to evaluate brivaracetam and levetiracetam synaptic vesicle glycoprotein 2A binding in healthy volunteers. Epilepsia. (2019) 60:958–67. 10.1111/epi.14701 30924924 PMC6532410

[14] Gillard M Fuks B Leclercq K Matagne A. Binding characteristics of brivaracetam, a selective, high affinity SV2A ligand in rat, mouse and human brain: relationship to anti-convulsant properties. Eur J Pharmacol. (2011) 664:36–44. 10.1016/j.ejphar.2011.04.064 21575627

[15] Biton V Berkovic SF Abou-Khalil B Sperling MR Johnson ME Lu S. Brivaracetam as adjunctive treatment for uncontrolled partial epilepsy in adults: a phase III randomized, double-blind, placebo-controlled trial. Epilepsia. (2014) 2014:57–66. 10.1111/epi.12433 24446953

[16] Hirsch M Hintz M Specht A. Schulze-Bonhage A.Tolerability, efficacy and retention rate of brivaracetam in patients previously treated with levetiracetam: a monocenter retrospective outcome analysis. Seizure. (2018) 61:98–103. 10.1016/j.seizure.2018.07.017 30118932

[17] Klein P Schiemann J Sperling MR Whitesides J Liang W Stalvey T. A randomized, double-blind, placebo-controlled, multicenter, parallel-group study to evaluate the efficacy and safety of adjunctive brivaracetam in adult patients with uncontrolled partial-onset seizures. Epilepsia. (2015) 56:1890–8. 10.1111/epi.13212 26471380

[18] St. Germaine-Smith C Liu M Quan H Wiebe SNJ. Development of an epilepsy-specific risk adjustment comorbidity index. Epilepsia. (2011) 52:2161–7. 10.1111/j.1528-1167.2011.03292.x 22004000

[19] Kröger K Küpper-Nybelen J Moerchel C Moysidis T Kienitz C Schubert I. Prevalence and economic burden of pulmonary embolism in Germany. Vasc Med. (2012) 17:303–9. 10.1177/1358863X12449363 22751745

[20] Weichle T Hynes DM Durazo-Arvizu R Tarlov E Zhang Q. Impact of alternative approaches to assess outlying and influential observations on health care costs. Springerplus. (2013) 2:614. 10.1186/2193-1801-2-614 24303338 PMC3843184

[21] Aícua-Rapún I André P Rossetti AO Ryvlin P Hottinger AF Decosterd LA. Therapeutic drug monitoring of newer antiepileptic drugs: a randomized trial for dosage adjustment. Ann Neurol. (2020) 87:22–9. 10.1002/ana.25641 31714640

[22] Ammar AA Ammar MA Owusu K Gilmore EJ. Intravenous brivaracetam for the management of refractory focal non-convulsive status epilepticus. BMJ Case Rep. (2020) 13:e234955. 10.1136/bcr-2020-234955 33229472 PMC7684825

[23] Ben-Menachem E Mameniškiene R Quarato PP Klein P Gamage J Schiemann J. Efficacy and safety of brivaracetam for partial-onset seizures in 3 pooled clinical studies. Neurology. (2016) 2016:314–23. 10.1212/WNL.0000000000002864 27335114 PMC4955277

[24] Chamberlain JM Kapur J Shinnar S Elm J Holsti M Babcock L. Efficacy of levetiracetam, fosphenytoin, and valproate for established status epilepticus by age group (ESETT): a double-blind, responsive-adaptive, randomised controlled trial. Lancet. (2020) 2020:1217–24. 10.1016/S0140-6736(20)30611-5 32203691 PMC7241415

[25] Fonseca E Guzmán L Quintana M Abraira L Santamarina E Salas-Puig X. Efficacy, retention, and safety of brivaracetam in adult patients with genetic generalized epilepsy. Epilepsy Behav. (2020) 102:106657. 10.1016/j.yebeh.2019.106657 31731108

[26] Moalong KM Espiritu AI Fernandez ML. Efficacy and tolerability of intravenous brivaracetam for status epilepticus: a systematic review. J Neurol Sci. (2020) 413:116799. 10.1016/j.jns.2020.116799 32278203

[27] Yates SL Fakhoury T Liang W Eckhardt K Borghs S D'Souza J. An open-label, prospective, exploratory study of patients with epilepsy switching from levetiracetam to brivaracetam. Epilepsy Behav. (2015) 52:165–8. 10.1016/j.yebeh.2015.09.005 26432008

[28] Szaflarski JP Sadek A Greve B Williams P Varner JA Moseley BD. Randomized open-label trial of intravenous brivaracetam versus lorazepam for acute treatment of increased seizure activity. Epilepsy Behav. (2020) 109:107127. 10.1016/j.yebeh.2020.107127 32417382

[29] Joseph JR Smith BW Williamson CA Park P. Seizure correlates with prolonged hospital stay, increased costs, and increased mortality in nontraumatic subdural hematoma. World Neurosurg. (2016) 92:366–70. 10.1016/j.wneu.2016.05.033 27237418

[30] Strein M Holton-Burke JP Smith LR Brophy GM. Prevention, treatment, and monitoring of seizures in the intensive care unit. J Clin Med. (2019) 8:1177. 10.3390/jcm8081177 31394791 PMC6722541

[31] Szaflarski JP DeWolfe JL. Levetiracetam use in the critical care setting. Front Neurol. (2013) 4:121. 10.3389/fneur.2013.00121 23986742 PMC3750522

